# Tofacitinib fails to prevent T cell transfer colitis in mice but ameliorates disease activity

**DOI:** 10.1038/s41598-023-30616-w

**Published:** 2023-03-07

**Authors:** Sudheendra Hebbar Subramanyam, Judit Turyne Hriczko, Angeliki Pappas, Angela Schippers, Nobert Wagner, Kim Ohl, Klaus Tenbrock

**Affiliations:** Department of Pediatrics, RWTH Aachen University, Pauwelsstr 30, 52074 Aachen, Germany

**Keywords:** Immunology, Gastroenterology, Rheumatology

## Abstract

Tofactinib is a JAK inhibitor approved for ulcerative colitis in humans. Despite of its’ proven effectiveness in humans, mechanistic data are scarce on the effectiveness of Tofactinib in experimental colitis in mice. We induced experimental colitis by transfer of CD4+CD25− isolated T cells into RAG2−/− (T and B cell deficient) mice and treated these mice with tofacitinib for 5–6 weeks either with a dosage of 10 or 40 mg/kg body weight immediately after CD4+ transfer or started treatment after first symptoms of disease for several weeks. While treatment with tofacitinib immediately after transfer resulted in an enhanced expansion of CD4+ T cells and did not prevent occurrence of colitis, treatment after start of symptoms of colitis ameliorated disease activity on a clinical basis and in histological analyses. Tofacitinib is effective in the treatment of murine experimental T cell transfer colitis, however does not prevent occurrence of disease.

## Introduction

The incidence of inflammatory bowel diseases (IBD) is rising and imposes a tremendous disease burden on the individual patient and also on society due to the often life-long need for immune suppression, involving high costs (biologicals) or severe side-effects (corticosteroids) and numerous days of sick leave. The exact etiology of the diseases is unknown, but involves multiple pathogenic factors including environmental changes, genetic predisposition, an abnormal gut microbiota and a broadly dysregulated immune system^1,2^. The therapy of ulcerative colitis (UC) is challenging. In spite of increasing therapeutic options, UC remains a disease with a high individual morbidity and relevant treatment costs. Moreover, reliable disease parameters for the guidance of treatment are lacking. Especially cytokines drive intestinal inflammation and pathologic processes associated with IBD. Janus kinases (JAKs) are central mediators of intracellular cytokine signaling and their blockade can interfere with more than 50 cytokines^3–5^. They are activated by extracellular cytokine receptors, which induces JAK-mediated receptor phosphorylation and facilitates activation of Signal Transducer and Activator of Transcription proteins (STATs), which then translocate to the nucleus and induce gene transcription^4,5^. It is therefore not surprising that dysregulations in JAK/STAT signaling are implicated in several inflammatory and autoimmune diseases^7^. The pan-JAK inhibitors including tofacitinib have been proven efficient in the treatment of UC and are approved for the treatment as second line option after failure of conventional disease modifying drugs. Despite this approval and despite the wealth of in vitro data on tofacitinib, surprisingly in vivo data on the effectiveness on colitis in mice are scarce. All published models are primarily models of chemically induced colitis (either DSS or oxazolone)^6–10^. One very recently publication included a humanized model^11^. To investigate the clinical effect of tofacitinib in mice, we performed a CD4+ transfer colitis into lymphopenic Rag2−/− mice and treated the mice with tofacitinib dissolved in the drinking water. Treatment with tofacitinib immediately after CD4+ transfer resulted in an enhanced expansion of inflammatory CD4+ T cells and did not prevent occurrence of colitis, however, treatment after start of symptoms of colitis ameliorated disease activity on a clinical basis and histological analyses.

## Results

### Low dose tofacitinib treatment does not prevent onset of colitis

**Figure 1:**
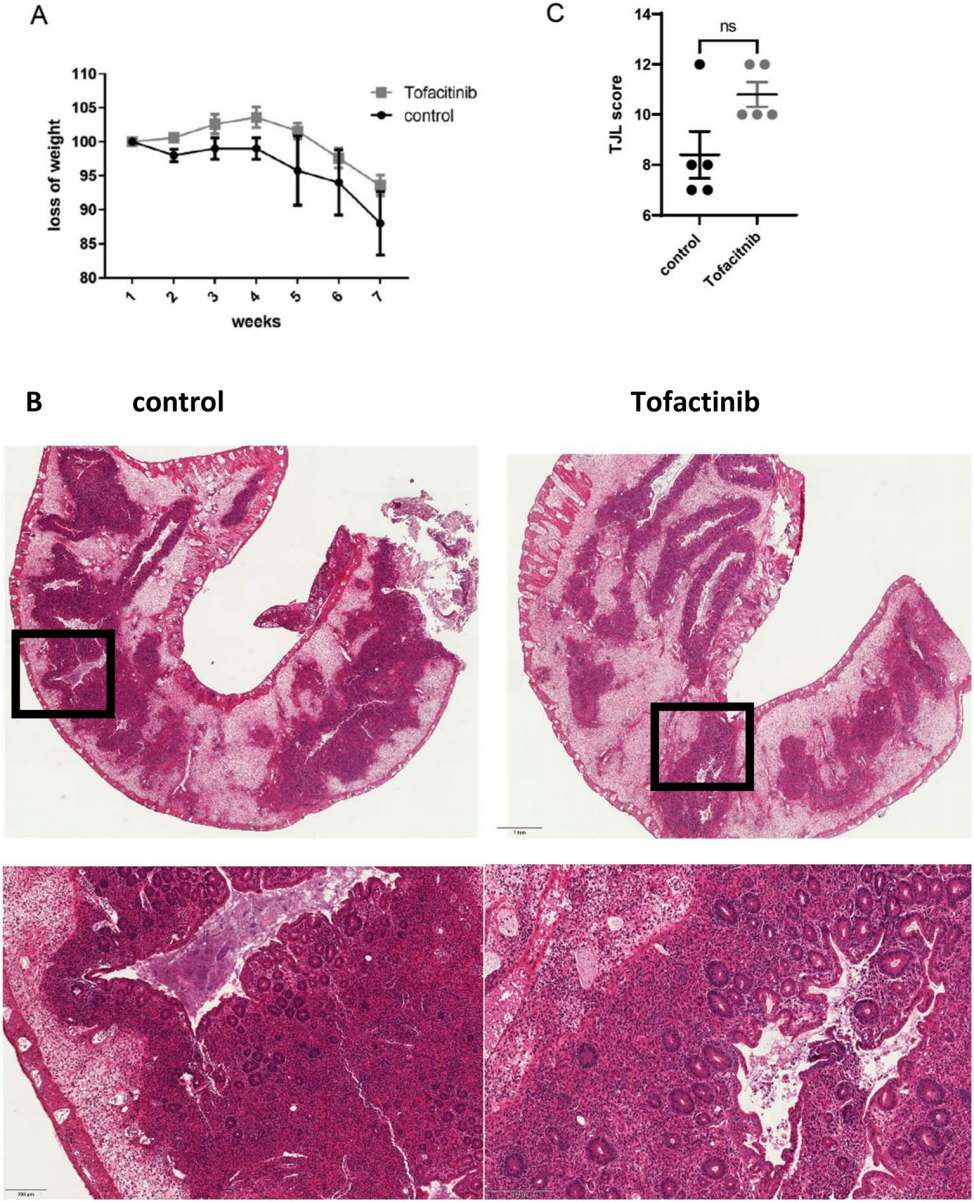
Low-doses of tofacitinib has no effect on disease activity in experimental colitis. Rag−/− mice were adoptively transferred with CD4^+^CD25-wild-type cells. Mice were weighed weekly and sacrificed 7 weeks after transfer. (**A**) Loss of body weight, two-tailed unpaired t-test were used to test for significance (one experiment with N = 4 controls, N = 5 Tofacitinib treated mice). (**B**) Representative histology of colon tissue (left: control, right: tofactinib), (**C**) Results of histological TJL (The Jackson Laboratory Score) scoring of colon sections.

**Figure 2:**
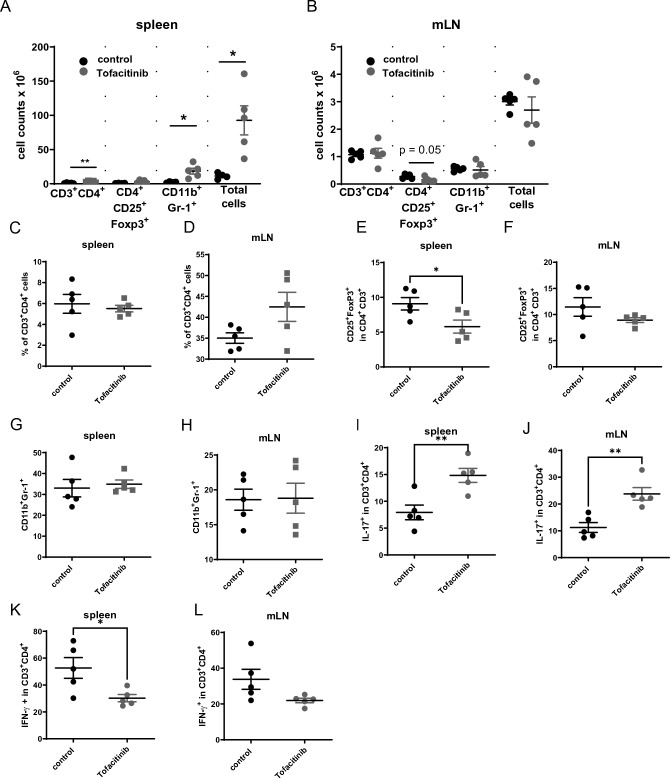
Low-doses of tofacitinib does not influence immune cell activation. (**A**) Absolute numbers of immune cells within the spleens assessed by flow cytometry (N = 4 controls, N = 5 Tofacitinib treated mice, two-tailed unpaired t-test were used). (**B**) Absolute numbers of immune cells within the mLNs assessed by flow cytometry (N = 5 controls, N = 5 Tofacitinib treated mice, p = 0.0084). Flow cytometric analysis was performed to determine percentages of (**C**) CD3+ CD4+ T cells within the spleen (** = 0.0084, * = 0.0121, * = 0.0120). (**D**) CD3+ CD4+ T cells within mLN. (**E**) % of CD25+ Foxp3+ in CD4+ CD3+ cells in the spleen (* = 0.034). (**F**) CD4+ CD3+ CD25+ Foxp3+ cells in the mLNs. (**G**) CD11b + Gr-1 + cells in the spleen. (**H**) CD11b + Gr-1 + cells within mLN. (**I**) IL17+ T cells within the spleen (** = 0.0066). (**J**) IL-17+ T cells within mLN (** = 0.0030). (**K**) IFN-γ+ T cells within the spleen (* = 0.0253). (**L**) IFN-γ+ T cells in mLN. In all data sets each dot represents one animal, error bars SEM, two-tailed unpaired t-tests were used to test for significance.

To induce transfer colitis, RAG2−/− mice were adoptively transferred with 2 × 10^6^ CD4^+^ CD25^-^ T cells. Initially, mice received 0.05 mg/ml tofacitinib in the drinking water, which results in a dosage of about 10 mg/kg/day bodyweight per day (low dose treatment). The control group received normal drinking water. As shown in Fig. 1A, onset and progression of weight loss did not differ between the group treated with tofacitinib and the untreated mice. In addition, colon histologies did not show any difference in the mice with and without treatment (Fig. 1B,C). Mice were sacrificed after 7 weeks, and flow cytometric and histological analyses were performed. Cell populations in the spleen and the mesenteric lymph nodes (mLNs) differed between treated and untreated mice. We found enhanced splenic numbers of CD3+ CD4+ cells as well as CD11b+ Gr1+ myeloid derived suppressor cells, while numbers in mLNs were not different except from decreased expression of Foxp3+ Tregs in tofacitinib treated animals (Fig. 2A,B). Regarding percentages of CD4+ cells, only Foxp3+ cells in the spleen were reduced, the rest showed no statistical differences (Fig. 2C–H). Regarding cytokine expression, percentages of interleukin 17 (IL-17) producing CD4+ T cells in spleens and mLNs of tofacitinib treated mice were enhanced, while percentages of IFN-γ producing CD4+ T cells was reduced (Fig. 2I–L). Summing up, low dose tofacitinib starting immediately after transfer during the expansion phase of CD4+ T cells enhances splenic numbers of CD3+ CD4+ T cells as well as of MDSCs and does not prevent colonic inflammation.

### High dose tofacitinib treatment does not prevent onset of colitis

**Figure 3:**
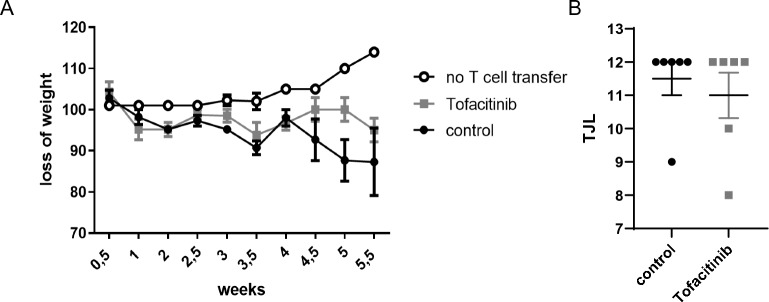
High-dose Tofacitinib treatment does not prevent weight loss and intestinal inflammation. Rag−/− mice were adoptively transferred with CD4^+^ CD25- wild-type cells Untreated Rag−/− mice were used as controls (**A**) Weight loss (N = 6 control mice, 6 Tofacitinib-treated mice and 4 mice without T cell transfer, one experiment). (**B**) Results of histological TJL (The Jackson Laboratory Score) scoring of colon sections.

**Figure 4:**
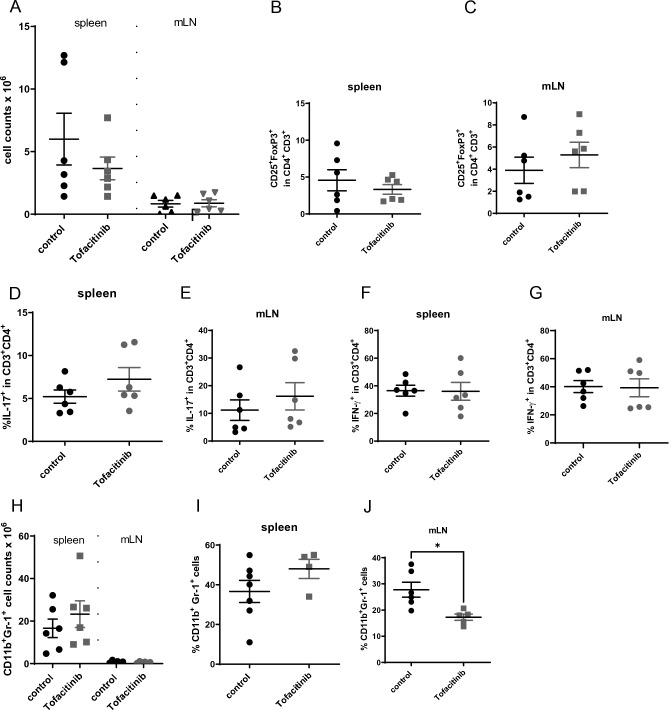
High-dose Tofacitinib treatment does not increase immune cell numbers. (**A**) Absolute numbers of immune cells within the spleens and mLNs assessed by flow cytometry. (**B**) Statistical analysis of CD4+ CD3+ CD25+ Foxp3+ cells in the spleen, N = 6 mice in each group). (**C**) Statistical analysis of CD4+ CD3+ CD25+ Foxp3+ cells in the mLNs, N = 6 mice in each group). (**D**) Statistical analysis of IL-17+ T cells within the spleen, N = 6 mice in each group). (**E**) Statistical analysis of IL-17+ T cells within mLNs, N = 6 mice in each group). (**F**) Statistical analysis of IFN-γ+ T cells within the spleen, N = 6 mice in each group). (**G**) Statistical analysis of IFN-γ+ T cells within mLNs, N = 6 mice in each group). (**H**) Absolute numbers of CD11b + Gr-1 + cells within the spleen and mLNs assessed by flow cytometry, N = 6 mice in each group. (**I**) Statistical analysis of CD11b + Gr-1 + cells within spleen, N = 6 control mice and N = 4 Tofactinib treated mice. (**J**) Statistical analysis of CD11b + Gr-1 + cells within spleen, N = 6 control mice and N = 5 Tofacitinib treated mice (* = 0.0112). For all data sets error bars indicate SEM, two-tailed unpaired t-tests were used to test for significance.

**Figure 5:**
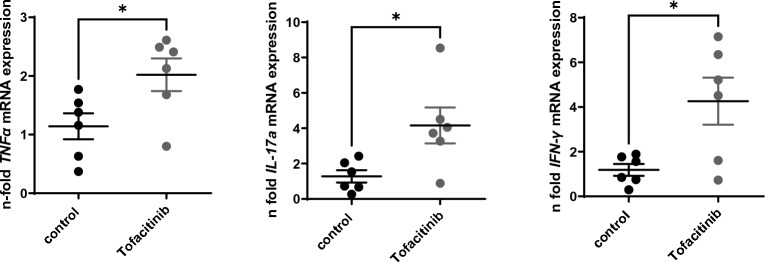
Inflammatory cytokines in the colon are enhanced by high dose Tofacitinib treatment. Expression of inflammatory cytokines analyzed by RT-qPCR, N = 6 mice in each group, two-tailed unpaired t-tests were used to test for significance (* = 0.0333, * = 0.0230, * = 0.0176).

After these rather unexpected results, we used a higher dosing regimen of tofacitinib. After adoptive transfer, mice received 0.2 mg/ml tofacitinib in the drinking water, which results in a dosage of about 40 mg/kg/day bodyweight per day (high dose treatment). Again, and comparable to the data in the low dose treated mice, tofacitinib treated mice developed disease and weight loss similar to control mice (Fig. 3A). Colon histology showed no statistical differences (Fig. 3B). The same was true for numbers and percentages of T cells including IFN-γ and IL-17 producing cells in spleen and mLN (Fig. 4A–G). Only expression of MDSCs was slightly different in terms of percentages of CD11b+ Gr-1+ cells showing more MDSCs in spleens of tofacitinib treated mice and less in the mLNs compared to untreated mice (Fig. 4H–J). We furthermore isolated RNA of whole colon and preformed real time PCR using primers for IL1β, IL-17, IFN-γ and TNFα. Colonic samples of tofacitinib treated mice showed higher expression of IL-17, IFN-γ and TNFα (Fig. 5A).

### High dose tofacitinib treatment ameliorates disease after onset of colitis

**Figure 6:**
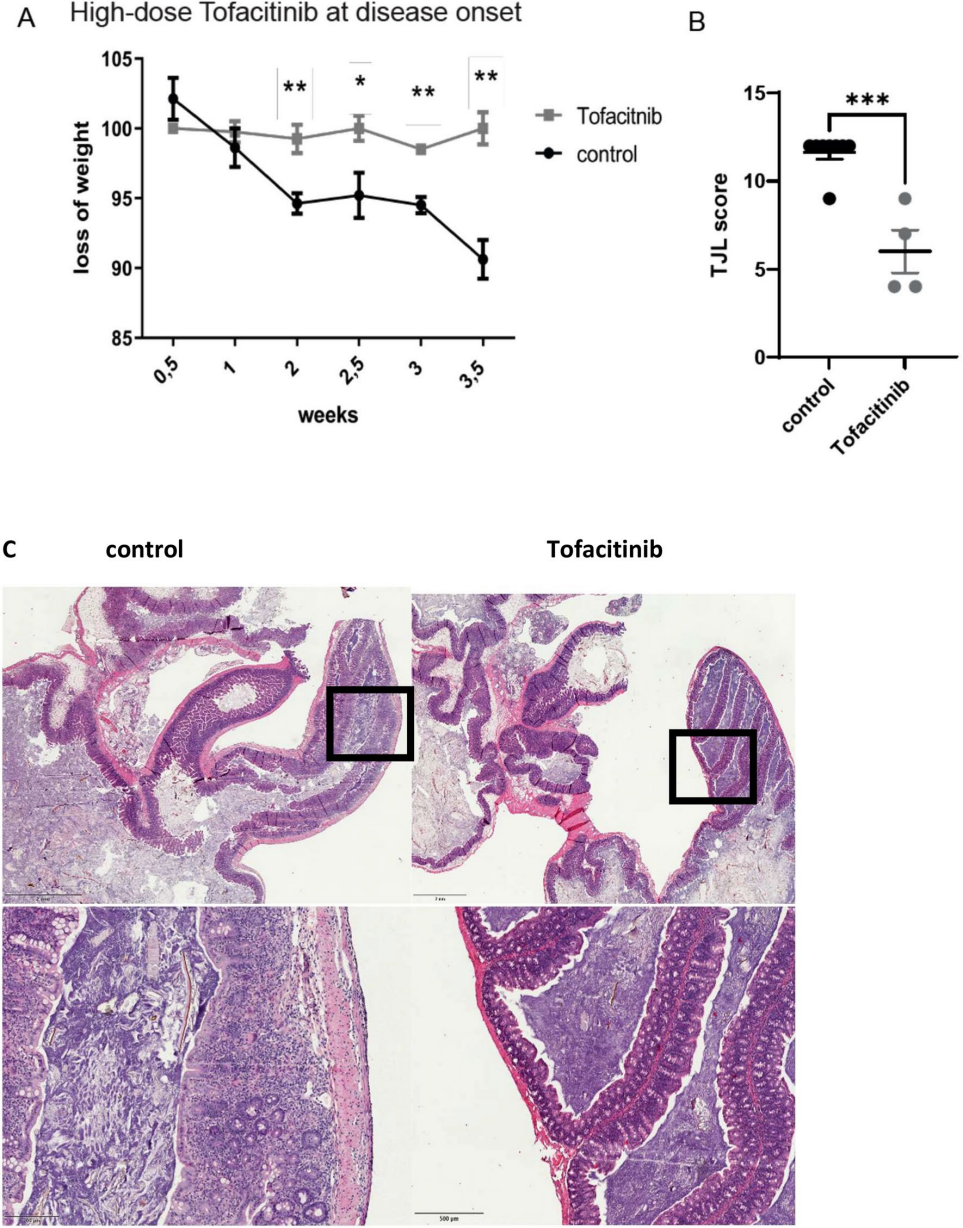
Tofacitinib treatment after onset of colitis reduces weight loss and intestinal inflammation. (**A**) Body weight as a percent of starting weight (N = 8 control mice, 4 Tofacitinib-treated mice, two-tailed unpaired t-tests were used, one experiment, ** = 0.0044, * = 0.0482, ** = 0.0011, ** = 0.0014). (**B**) Results of histological TJL (The Jackson Laboratory Score) scoring of colon sections. (**C**) Representative histology of colon tissue (left: control, right: tofactinib).

**Figure 7:**
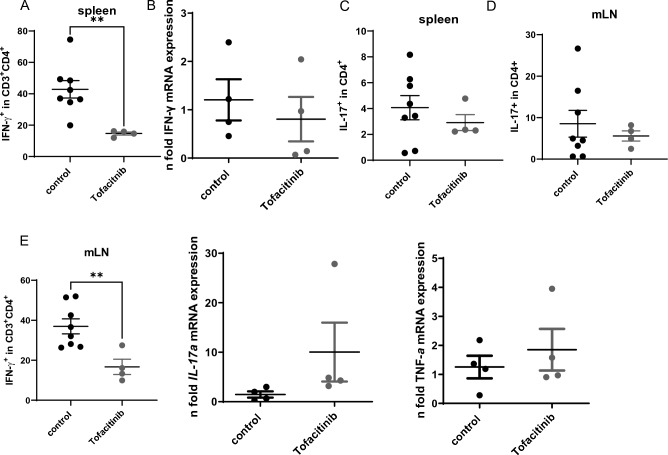
Tofacitinib treatment after onset of colitis reduces IFN-γ secretion. (**A**) Statistical analysis of IFN-γ+ T cells within the spleen, (N = 8 control mice and N = 4 Tofacitinib treated mice, ** = 0.0060)). (**B**) Statistical analysis of IFN-γ+ T cells within mLNs, (N = 8 control mice and N = 4 Tofacitinib treated mice, ** = 0.0070). (**C**) Statistical analysis of IL-17+ T cells within the spleen, (N = 8 control mice and N = 4 Tofacitinib treated mice). (**D**) Statistical analysis of IL-17+ T cells within mLNs, (N = 8 control mice and N = 4 Tofacitinib treated mice. (**E**) Expression of inflammatory cytokines analyzed by RT-qPCR, N = 4 c mice in each group. For A-C, error bars indicate SEM, two-tailed unpaired t-tests were used to test for significance.

We next decided to adopt a “treatment” regime, which started after onset of first symptoms defined by loss of weight in the first mouse. This was around 2 weeks after adoptive transfer. We again supplied the mice with 0.2 mg/ml tofacitinib in the drinking water, which represented a dosage of about 40 mg/kg/day bodyweight. This treatment protocol resulted in a stabilization of the bodyweight in tofacitinib treated mice (Fig. 6A) and in an amelioration of disease with lower inflammation in the histologic colon samples (Fig. 6B,C). Moreover, percentages of IFN-γ producing T cells in spleens and mLNs were reduced in the tofacitinib treated mice (Fig. 7A,B), while there was no difference in the percentages of IL-17 producing T cells (Fig. 7C,D). Numbers of CD11b+ Gr1+ myeloid derived suppressor cells were comparable in both groups (not shown) as well as cytokine mRNA expression (Fig. 7E).

## Discussion

Our data demonstrate that treatment with tofacitinib has different effects on the clinical outcome of T cell transfer colitis depending on the onset of treatment. Treatment that starts immediately after T cell transfer does not prevent disease. If anything, it even results in aggravation of disease represented by colonic histology and expression of inflammatory cytokines in T cells and in colonic RNA. If treatment is started after the onset of first symptoms, tofacitinib ameliorated disease shown by weight stabilization, colonic histology and decreased production of inflammatory cytokines.

Our findings of the therapeutic effect of tofacitinib were somehow expected and go in line with a recent paper from Lechner et al., which described effectiveness in the treatment of oxazolone induced colitis with downregulation of T cell derived cytokines like IL-5, IL-6, IL-9, IL-13 and IL-17A^7^. This colitis model is mediated by a hypersensitivity reaction related to oxazolone after sensitization and therefore primarily CD8 mediated. In addition, tofacitinib exerts a similar effect on human organoids from UC and control endoscopies, where tofacitinib reduces inflammatory cytokines by inhibition of pSTAT1/3 expression in T-cells^12^. In vitro experiments indicated that the effects of tofacitinib were mediated through suppression of IL-17 and IFN-γ production and proliferation of CD4 T cells, presumably Th1 and Th17. In an autoimmune lymphoproliferative (ALPS) mouse model, tofactinib treatment decreased expression of TCRαβ(+)CD4(-)CD8(-)T lymphocyte numbers while it increased CD8( +) T cells without a remarkable effect on CD4(+) lymphocytes including FoxP3(+) regulatory T cells^13^.

We were surprised to find no effect on colonic inflammation and even an expansion of CD4+ T cell numbers in the mice that were treated directly after adoptive transfer of T cells. This is in contrast to CD4+ T-cell-mediated murine acute graft-versus-host disease, which can be prevented by tofacitinib. This is related to inhibition of the interferon-γ pathway^14^. It has been shown before that human T-lymphocyte proliferation is inhibited by tofacitinib, however antiproliferative effects are strongly attenuated, when tofacitinib is given to preactivated PBMCs, which suggests that the surrounding conditions play a pivotal role^15^. Moreover, and again in humans, during phase III studies in patients with rheumatoid arthritis with more than 7000 patients, tofacitinib treatment resulted in an initial increase in lymphocyte counts within the first 3 months versus pretreatment baseline, which gradually declined to steady state by ~ 48 months^16^. In addition, it was shown before that low dose tofacitinib accelerates the onset of experimental autoimmune encephalomyelitis by potentiating Th17 differentiation^17^. This goes in line with our first experiment, in which we also found an enhancement of IL-17 producing cells. Thus, apart from timing also dosage might play a critical role. However, other publications suggest that tofacitinib inhibits proliferation in CD4+ and CD8 + T cells as well as Th1 and Th17 differentiation in vitro, while Th2 and regulatory T cell lineages remained unaffected^6^. Moreover tofacitinib is efficacious in patients with active UC but not Crohn’s disease (CD), which can be explained that JAK/STAT molecules play pleiotropic and cell specific roles^18,19^. Considering myeloid suppressor cells, one of our primary assumptions was that these immune-modulating cells might play a role in control of colitis, since tofacitinib facilitates the expansion of MDSCs and ameliorates experimental arthritis as well as interstitial lung disease in SKG mice^20,21^. In line with that paper, GR1 + CD11b + MDSCs were also expanded in Rag2−/− mice during early treatment with tofactinib in our hands, however, their numbers did not affect disease outcome. The data on MDSCs in inflammatory bowel disease are conflicting. Haile et al. as well as Zhang et al. described and anti-inflammatory phenotype of MDSCs^22,23^ while Guan et al. as well as a recent paper from de Cicco described pro-inflammatory properties depending on the disease model^24,25^. Therefore the effect of MDSCs in disease control remain uncertain. Macrophage differentiation is affected by tofacitinib^8^ suggesting a switch from M1 to M2 type macrophages. Strikingly, and comparable to our data, DSS-colitis development was not prevented in that study, but treatment was effective as soon as signs of disease developed in a rescue model of DSS colitis.

Summing up, tofacitinib is effective in the treatment of experimental CD4+ T cell transfer colitis in mice while it does not prevent occurrence of colitis. Thus time-point of treatment plays a significant role.

## Methods

### Mice strains

Experiments were performed with 8–12 week old C57BL/6 mice. RAG−/− (C57BL/6 J) mice were provided by A. Schippers. We used mixed genders. All mice were bred in our animal facility and kept under standardized conditions. All methods used in mice were carried out in accordance with ARRIVE guidelines.

### Cell isolation

Single cell suspensions were isolated from spleens and erythrocytes were lysed with lysis buffer. CD4^+^CD25^+^ Treg cell isolation kits (Miltenyi) were used to isolate CD4^+^CD25^-^ cells and perform adoptive transfer colitis.

### Transfer colitis

To induce transfer colitis, RAG2−/− mice were adoptively transferred with 2 × 10^6^ CD4^+^ CD25^-^ T cells. Animals were sacrificed as soon as the loss of weight exceeded 10% of the initial bodyweight. Spleens and mLNs were harvested for further analysis. One part of the colon was fixed in formalin for histological scoring (caecum) and the upper part was fixed in RNAlater (Qiagen, Germany) for subsequent mRNA analysis.

For soluble Tofacitinib treatment, mice received either 0.05 mg/ml dissolved in drinking water resulting in a dosage of 10 mg/kg/day of Tofacitinib or of 0.2 mg/ml dissolved in drinking water resulting in a dosage of 40 mg/kg/day of Tofacitinib until end of experiments. We adopted this method from Frederike Berberich-Siebelt, who showed that tofacitinib is soluble and stable in drinking water and is taken up by mice to sufficient amounts (unpublished, personnel communication). A treatment scheme is shown in Supplementary Fig. 1.

### Histological scoring

4 µm paraffin sections from the fixed colon (caecum and proximal part) were cut serially, mounted onto glass slides, and deparaffinized. The colon sections were stained with hematoxylin and eosin by the Core Facility (IZKF) of the RWTH Aachen University. Blinded histological scoring was performed using a standard microscope, based on TJL method as described previously^26^. Each colon section was scored for the four general criteria: severity, degree of hyperplasia, degree of ulceration, if present, and percentage of area involved. A subjective range of 1–3 (1 = mild, 2 = moderate, 3 = severe) was used for the first three categories. Severity: Focally small or widely separated multifocal areas of inflammation limited to the lamina propria were graded as mild lesions (1). Multifocal or locally extensive areas of inflammation extending to the submucosa were graded as moderate lesions (2). If the inflammation extended to all layers of the intestinal wall or the entire intestinal epithelium was destroyed, lesions were graded as severe (3). Hyperplasia: Mild hyperplasia consisted of morphologically normal epithelial lining that was at least twice as thick (length of crypts) as adjacent or control mucosa. Moderate hyperplasia was characterized by the epithelial lining being two- or three-times normal thickness, cells were hyperchromatic, numbers of goblet cells were decreased, and scattered individual crypts developed an arborizing pattern. Severe hyperplastic regions exhibited markedly thickened epithelium (four or more times normal thickness), marked hyperchromasia of cells, few to no goblet cells, a high mitotic index of cells within the crypts, and numerous crypts with arborizing pattern. Ulceration was graded as: 0 = no ulcer, 1 = 1–2 ulcers (involving up to a total of 20 crypts), 2 = 1–4 ulcers (involving a total of 20–40 crypts), and 3 = any ulcers exceeding the former in size. A 10% scale was used to estimate the area involved in the inflammatory process. 0 = 0%, 1 = 10–30%, 2 = 40–70%, 3 =  > 70%.

### Flow cytometry

For surface staining, single cell suspensions were stained with anti-CD4, anti-CD3, (from Invitrogen and eBioscience, Germany). For intracellular staining of Foxp3, cells were fixed and permeabilized with a FOXP3 staining buffer set (eBioscience, Germany) following the manufacturer’s instructions and stained with respective antibodies (Invitrogen) for 30 min. Intracellular cytokines were stained with anti- IFN-γ APC (Invitrogen) and anti-IL-17-Alexa 488 (BD), after PMA (30 nM) and Ionomycin (1.5 µM) (both Sigma-Aldrich, USA) restimulation in the presence of Golgi Plug for 5 h (antibodies see Table 1). Representative gating strategies and dot plots are shown in Supp Fig. 2.

**Table 1:** Antibodies used in the experiments.

Antibody	Lot Number	Company	Clone
CD11b Pacific Blue	4329941	Invitrogen	M1/70
Gr1 FITC	2233545	BD Biosciences	1 A8
CD3 APC	2293305	Invitrogen	145-2C11
CD4 PE	E01009-1635	eBioscience	GK1.5
CD4 Pacific Blue	2261418	Invitrogen	RM4-5
IFN-γ APC	2011141	Invitrogen	XMG1.2
IL-17 Alexa 488	7096813	BD Biosciences	TC11-18H10
CD25 Pacific Blue	2055195	Invitrogen	PC61.5
Foxp3 FITC	2152040	Invitrogen	FJK-16 s

### RNA isolation and quantitative real-time PCR

Total RNA from colon tissue (distal part and sigma) was isolated using the RNeasy Mini Kit (Qiagen, Germany). cDNA was then generated from 200 ng total RNA using the RevertAid H Minus First Strand cDNA Synthesis Kit (Thermo Fisher Scientific, USA) according to the manufacturer’s instructions. RT-PCR was performed using the SYBR Green PCR kit (Eurogentec, Germany) and data were acquired with the ABI prism 7300 RT-PCR system (Applied Biosystems/Life Technologies, Germany). Each measurement was set up in duplicate. After normalization to the endogenous reference control gene β-actin for mice, the relative expression was calculated (for primer sequences see Table 2).

**Table 2:** Sequences of primers used for RT PCR.

Gene	Forward primer (5′ → 3′)	Reverse primer (5′ → 3′)
IFN-γ	TGCCAAGTTTGAGGTCAACAACCCA	CCCACCCCGAATCAGCAGCG
IL-17A	AGCTGGACCACCACATGAATT	CCACACCCACCAGCATCTTC
TNF-α	AGAAACACAAGATCCTGGGACAGT	CCTTTGCAGAACTCAGGAATGG

### Statistical analysis

All data are presented as mean ± standard error (SEM). Differences between two groups were evaluated using two-tailed unpaired or paired (if indicated), Student’s t-test. All statistical analysis and subsequent graphics generation were performed using GraphPad Prism version 9.0 (GraphPad Software, USA). A *p* value < 0.05 was statistically significant.

### Study approval

The study was approved by the regional government authorities and animal procedures were performed according to German legislation for animal protection. Permission for the projects was granted by the Regierungspräsident/LANUV Nordrhein-Westfalen.

## Supplementary Information

Supplementary Information.

## Data Availability

All data generated or analysed during this study are included in this paper and in the supplementary information files.
