# Author Correction: Tofacitinib fails to prevent T cell transfer colitis in mice but ameliorates disease activity

**DOI:** 10.1038/s41598-025-97999-w

**Published:** 2025-04-16

**Authors:** Sudheendra Hebbar Subramanyam, Judit Turyne Hriczko, Angeliki Pappas, Angela Schippers, Nobert Wagner, Kim Ohl, Klaus Tenbrock

**Affiliations:** Department of Pediatrics, RWTH Aachen University, Pauwelsstr 30, 52074 Aachen, Germany

Correction to: *Scientific Reports* 10.1038/s41598-023-30616-w, published online 07 March 2023

The original version of this Article contained errors in the Author Contributions section, where

“S.H.S.: performed experiments and analysis of data and wrote the paper, J.T.H., performed experiments, K.O. performed experiments, analysis of data, wrote paper, A.P.: analysis of data, wrote the paper, A.S.: analyzed data, wrote the paper, N.W.: wrote the paper, K.T.: designed the study, analyzed data, wrote the paper.”

now reads:

“S.H.S.: performed experiments, analyzed data, and wrote the manuscript, J.T.H.: performed experiments, K.O.: performed experiments, analyzed data, and revised the manuscript, A.P.: analyzed the data and reviewed the manuscript, A.S.: analyzed data and reviewed the manuscript, N.W.: reviewed the manuscript, K.T.: designed the study, analyzed data, and revised the manuscript.”

The original Article has been corrected.

