# Supplementary material for: Tofacitinib fails to prevent T cell transfer colitis in mice but ameliorates disease activity
Source: Sci Rep. 2023 Mar 7;13:3762. doi: 10.1038/s41598-023-30616-w (PMC9992375; doi:10.1038/s41598-023-30616-w)
Supplement: Supplementary file 1 — Supplementary Information. [file 41598_2023_30616_MOESM1_ESM.docx]

**
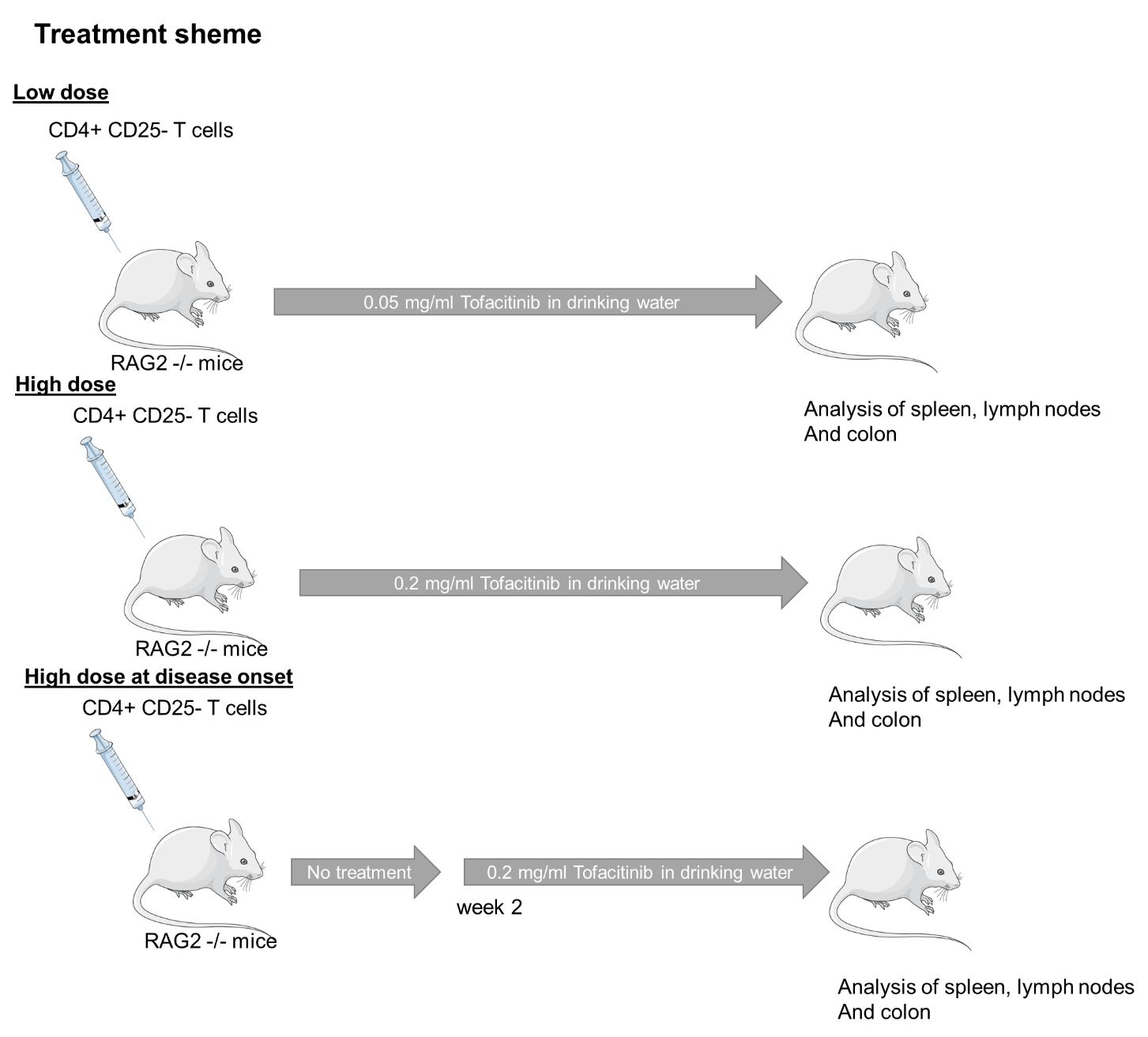
Supplemental Figures**

**Suppl. Fig. 1: Treatment scheme**

**
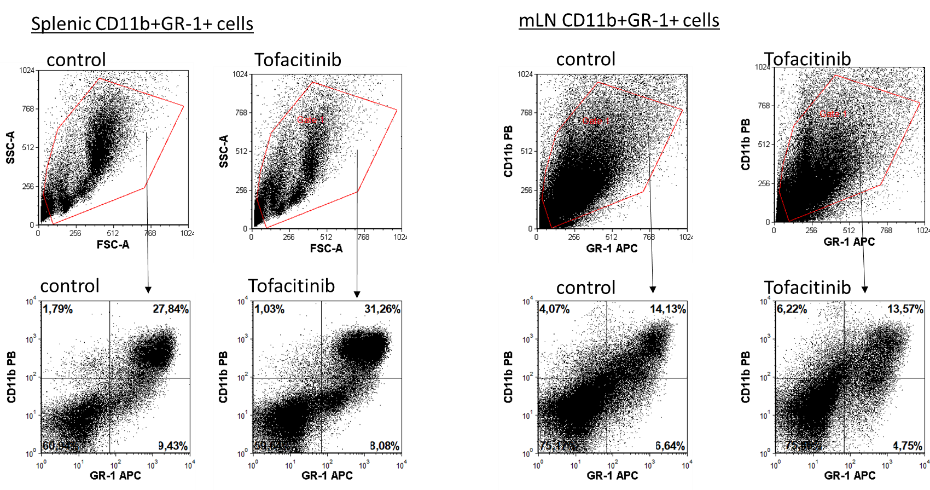
**

**
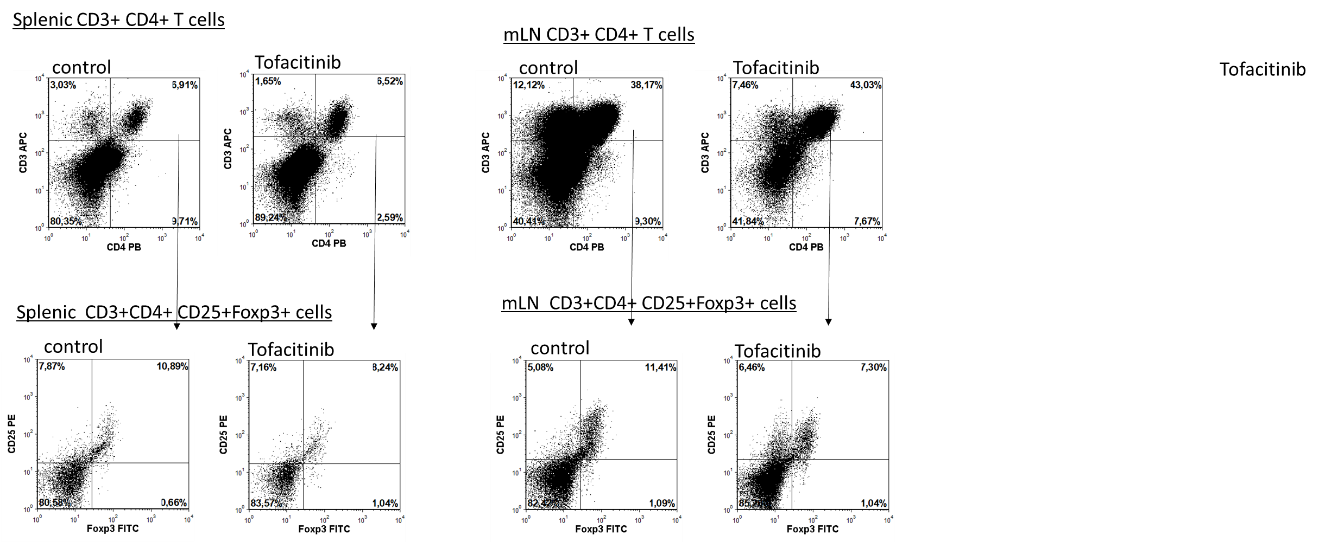
**

**
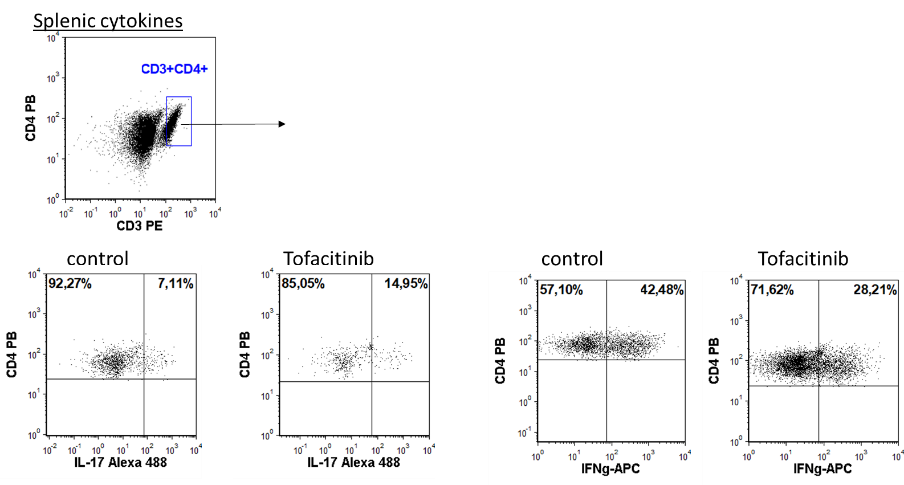

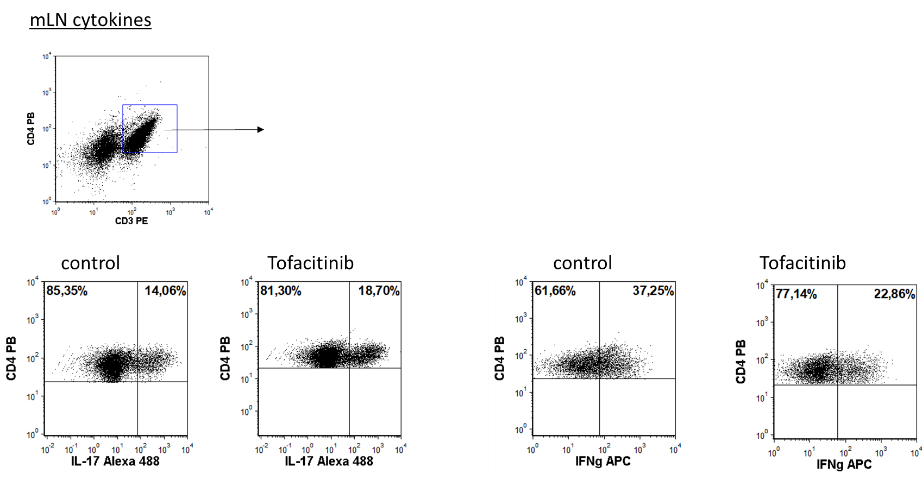
**

**Suppl. Fig. 2: Gating strategies and representative Dot plots.**
